# Is Subjective Age Associated with Physical Fitness in Community-Dwelling Older Adults?

**DOI:** 10.3390/ijerph19116841

**Published:** 2022-06-03

**Authors:** Jin Wang, Jiabin Yu, Xiaoguang Zhao

**Affiliations:** 1 Faculty of Sport Science, Ningbo University, Ningbo 315211, China; wangjin@nbu.edu.cn (J.W.); yujiabin@nbu.edu.cn (J.Y.); 2 Research Academy of Grand Health, Ningbo University, Ningbo 315211, China

**Keywords:** subjective age, physical fitness, physical activity, older adults, health outcomes

## Abstract

Although subjective age has been associated with a range of health-related outcomes, there has been little systematic study on the relationship between the subjective age and physical fitness in a given population. The purpose of this study was to determine the prospective association between subjective age and physical fitness in community-dwelling older adults. A sample of 276 older people who lived in the community was studied. Subjective age was measured by a face-to-face interview. Grip strength, balancing on one leg with eyes open, the 30 s chair stand test, 4 m habitual walk, and 6 min walk test were measured to reflect physical fitness. Results indicated that the felt younger older adults had a higher level of physical fitness compared to their felt older and felt the same counterparts. Multiple linear regression analysis indicated that all the measured physical fitness items were significantly associated with subjective age in older men. All of the measured physical fitness items except for the 4 m habitual walk were remarkably related to subjective age in older women. The findings suggest that subjective age is closely associated with physical fitness in community-dwelling older adults. Much attention should be paid to the promotion of physical fitness to improve the subjective age of older adults.

## 1. Introduction

Aging is an unavoidable physiological process. Chronological age and subjective age are considered to be two important concepts and are frequently used as proxies for aging. In the scientific community, chronological age has been generally utilized when dividing individuals into different age groups [1,2]. However, in the gerontology field, chronological age seems to be less important than it is projected to be, especially for the oldest adults. This is because even if persons have the same chronological age, their degree of aging and overall health may differ from person to person, affected by factors such as familial heredity, diseases, physical activity, and eating habits [3,4,5]. Thus, chronological age is not a perfect measure for assessing one’s degree of aging and overall health.

Subjective age, or felt age (how young or old a person feels relative to chronological age), is another concept that affects aging. Subjective age has attracted growing attention in the last decade since it is shown to be related to a range of health-related outcomes in older people [6]. A great number of studies have documented that older subjective age is consistently associated with worse mental and cognitive health [7,8], poor sleep quality [9], lower overall functioning [10,11], and higher morbidity and mortality [12,13]. Furthermore, subjective age has been found to mediate the association between aging and a range of health-related outcomes [14,15]. It has been reported that subjective age, as a biopsychosocial marker of aging, can predict a series of psychological and physical health outcomes [16,17]. Although subjective age has been associated with a range of health-related outcomes, to our knowledge, there has been little systematic study on the relationship between subjective age and physical fitness in a given population.

There are reasons why we expected that an association exists between subjective age and physical fitness in older adults. It is widely accepted that physical fitness has a range of physical, psychological, and cognitive benefits that have also been found to be associated with subjective age in older adults. Specifically, older adults with a higher level of physical fitness generally have better mental and cognitive health [18,19], lower incidence of chronic diseases [20], higher quality of life [21], and lower morbidity and mortality [22]. These health-related outcomes have been reported to more or less relate to subjective age in older adults. Moreover, although there are no systematic studies on physical fitness, two previous studies have proven, respectively, that older people with a younger subjective age generally have a faster walking speed and a greater grip strength score than those with an older subjective age [23,24]. The major limitation of these studies is that only one measure was selected to reflect physical function or physical fitness.

Although it is reasonable to postulate that physical fitness is related to subjective age, how many and how well physical fitness components are linked to subjective age in a given population are not clear. Therefore, the present study aimed to determine the relationship between subjective age and physical fitness, and figure out which aspects of physical fitness components are closely related to subjective age in community-dwelling older adults. It is hypothesized that a younger subjective age would be associated with a higher level of physical fitness in community-dwelling older adults.

## 2. Materials and Methods

### 2.1. Participants

We carried out this cross-sectional study during the period from March 2019 to 31 July 2019 in Ningbo city in China. Participants were recruited in the study through community health service centers close to our university by adopting a convenience sampling method. Our participants came from different communities and had to comply with the study criteria. The inclusion criteria for our study were as follows: participants (1) aged 65 years and older; (2) lived in communities; (3) were able to accomplish all the physical fitness tests; and (4) agreed to participate in the study. The exclusion criteria for the study were as follows: participants (1) had cardiovascular diseases such as myocardial infarction, stroke, and coronary heart disease, and (2) had musculoskeletal diseases including joint and muscle pain and lower limb arthritis, which impact physical activities.

**Figure 1:**
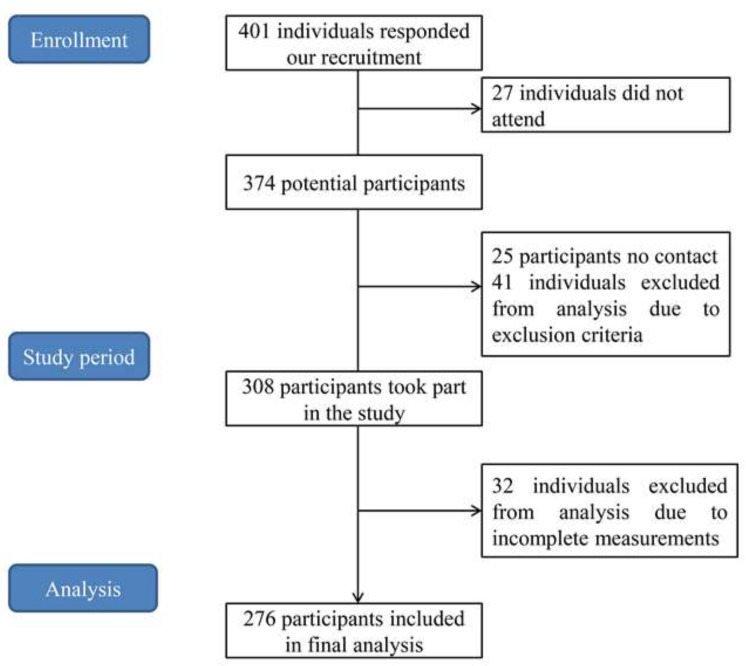
The flow diagram of the participants inclusion.

A flow diagram of the participant’s inclusion is shown in Figure 1. At first, 401 older adults responded to our recruitment, but 27 of them did not attend, 25 older adults were out of touch, and 41 and 32 older adults were excluded from the analysis, respectively, because of the exclusion criteria and incomplete tests. As a result, the study was carried out with the participation of 276 community-dwelling older adults. Before the test, the study’s purposes and procedures were explained to each participant by an experienced researcher (a sports medicine physician) and then the participant was requested to sign a written informed consent form. The research protocol was reviewed and approved by the Human Ethics Board of Ningbo University (approval number: RAGH2019011601).

### 2.2. Procedures

All of the measurements were conducted on the same day (9:00 a.m. to 5:00 p.m.) in two phases. In the first phase, the subjective age was collected for each participant through a face-to-face interview by an experienced researcher. The measurements for the subjective age involved in our study have been detailed in previous studies [11,15,16,17]. In the second phase, the physical fitness was reflected by assessing the muscle strength, balance, muscle endurance, usual gait speed, and cardiopulmonary endurance. Referring to previous studies [4,5,25,26], a series of test items including grip strength, balancing on one leg with eyes open, the 30 s chair stand test, 4 m habitual walk, and 6 min walk test were selected in our study. Before the test, all participants conducted a 5 min warm-up and were then familiarized with the different test items. The test items above-mentioned were measured by trained testers in a random order. The entire measurements for each participant took approximately one hour.

### 2.3. Subjective Age Measurement

Subjective age, or felt age, was measured by asking the participants to report how old they felt in years at the time of the face-to-face interview. According to previous studies [15,16,17], a proportional age discrepancy score, which is calculated by subtracting the chronological age from the felt age and dividing by the chronological age, is used frequently to indicate subjective age. A positive score of the proportional age discrepancy represents the participants with a younger subjective age than their chronological age, while a negative score represents the participants with an older subjective age compared to their chronological age. In this study, the participants were divided into three groups: the felt younger group (subjective age < chronological age), felt the same group (subjective age = chronological age), and felt older group (subjective age > chronological age) based on their proportional age discrepancy scores.

### 2.4. Physical Fitness Measurement

Grip strength: A dynamometer (Grip-D5101, Takei, Japan) was used to assess the grip strength. Participants were asked to hold the dynamometer in their preferred hands with arms down the sides of their body, and then clenched the handle with maximum force. There were two trials for this test with a 30 s rest between trials. The maximum reading to the nearest kilogram (kg) was recorded for data analysis.

Balancing on one leg with eyes open: Participants were requested to maintain body balance with their eyes open and hands touching the waist. During the tests, the participants were asked to use their preferred foot to stand and the other foot left the ground. The record was the number of seconds (s) between the time of the non-preferred foot left the ground and the time that balance was lost. Two trials were given, and the maximum number of seconds was used for further analysis.

The 30 s chair stand test: Participants were asked to sit on a chair (45-cm height without armrests) until their feet were placed on the ground with their arms crossed in front of their chest. During the tests, the participants were requested to stand up until their hip and knee were extended fully, and then sit down. The participants were told to repeat this movement as fast as possible for 30 s. There were two trials for this test, and the maximum number of repetitions within 30 s (repetitions/30 s) was recorded for data analysis.

The 4 m habitual walk: There was a 10 m straight course for this test. Participants were asked to walk at their habitual speed on the straight course. To minimize the impact of acceleration phases, we measured the number of seconds (s) between the time of the 3-m mark and the 7-m mark during the tests. Two trials were given, and the minimum number of seconds was used for further analysis.

The 6 min walk test: Before the test, the participants were asked to sit on chairs to rest for about 5 min at the start area, and they were asked to walk but not run for 6 min. During the tests, the testers informed the participants of the consumed time in each minute and encouraged them to continue walking as fast as possible. After the test, we measured the meters (m) of the walking distance for every participant. Only one trial was given for this test.

### 2.5. Statistical Analysis

We used the descriptive statistics to present an overview of the dataset. All of the measured subjective age and physical fitness parameters are shown as the means with standard deviations. The normality of the variables was evaluated using the Shapiro–Wilk test. One-way analysis of variance was employed to compare the differences in the physical fitness items among three groups, namely, the felt younger group, felt the same group, and felt older group. A Bonferroni post-hoc test was performed to compare the differences between the different groups. Multiple linear regression analysis (enter) was employed to obtain the main physical fitness components that were significantly associated with subjective age in community-dwelling older adults. The age discrepancy score was set as the dependent variable and the physical fitness items were set as independent variables. The analysis of data was executed using the IBM Statistical Package for Social Sciences software (SPSS version 25.0; SPSS Inc., Chicago, IL, USA), and the level of the statistically significant difference was set at *p* < 0.05.

## 3. Results

A total of 94 older men and 182 older women in the community were involved in the final analysis. The basic characteristics of the participants are presented in Table 1. The mean chronological age was 71.21 ± 5.04 and 70.71 ± 4.82 years, the mean subjective age was 69.09 ± 7.77 and 69.85 ± 7.11 years, and the mean age discrepancy score was 0.03 ± 0.06 and 0.01 ± 0.05 for older men and women, respectively. It was also found that older men had a better physical fitness than older women.

For older men, there were statistical differences in all of the measured physical fitness items among the felt younger group, felt the same group, and felt older group (*p* < 0.01). The post hoc test showed significant differences in all the measured physical fitness items between the felt younger group and the felt older group, significant differences in all the measured physical fitness items except for balancing on one leg with eyes open between the felt younger group and felt the same group (*p* < 0.05), and no significant differences in all the measured physical fitness items between the felt the same group and felt older group (Table 2); for older women, there were statistical differences in all of the measured physical fitness items among the felt younger group, felt the same group, and felt older group (*p* < 0.05). The post hoc test indicated significant differences in all the measured physical fitness items between the felt younger group and the felt older group, significant differences in all of the measured physical fitness items except the 4-m habitual walk between the felt younger group and felt the same group (*p* < 0.05), and no significant differences in all of the measured physical fitness items except for the 6-min walk test between the felt the same group and felt older group (Table 2).

The results of the multiple linear regression analysis between the age discrepancy score and physical fitness items can be seen in Table 3. For older men, all of the measured physical fitness items were the main components that were significantly associated with the subjective age (*p* < 0.05). The explanatory power of subjective age formed by grip strength, balancing on one leg with eyes open, 30 s chair stand test, 4 m habitual walk, and 6 min walk test was 85% (F = 46.35, *p* < 0.001). For older women, all of the measured physical fitness items except for the 4 m habitual walk were the main components that were remarkably related to subjective age (*p* < 0.05). The explanatory power of the subjective age developed by grip strength, balancing on one leg with eyes open, 30 s chair stand test, 4 m habitual walk, and 6 min walk test was 54% (F = 41.16, *p* < 0.001).

## 4. Discussion

This is the first cross-sectional research, to the best of our knowledge, to systematically study the relationship between subjective age and physical fitness, and find out which aspects of the physical fitness components are closely related to subjective age in a given population. The current study showed that the felt younger older adults had a higher level of physical fitness compared to their felt older and felt the same counterparts. Further analysis indicated that all of the measured physical fitness items including grip strength, balancing on one leg with eyes open, the 30 s chair stand test, 4 m habitual walk, and 6min walk test were significantly associated with subjective age in older men. All of the measured physical fitness items except for the 4 m habitual walk were remarkably related to subjective age in older women. The findings suggest that subjective age is associated with physical fitness in community-dwelling older adults.

Muscular strength is one of the most important components of physical fitness and is frequently assessed by grip strength. It has been found that grip strength is an independent predictor for a range of health-related outcomes such as bone mineral density [27], all-cause mortality, and cardiovascular diseases [28]. Moreover, Stephan and collaborators [24] reported that grip strength was closely associated with subjective age in older persons where the result was similar to our findings. In our study, the felt younger older adults were found to have a greater grip strength compared to their felt older and felt the same counterparts. The multiple linear regression analysis showed that the grip strength was significantly related to subjective age. Given the above, these findings suggest that muscle strength is an important physical fitness component that is closely linked to subjective age in community-dwelling older adults.

In the present study, body balance and muscle endurance were reflected by balancing on one leg with eyes open and the 30 s chair stand test, respectively. Our results revealed that body balance and muscle endurance are significantly related to subjective age in older adults. In previous studies, although there was no direct evidence, indirect evidence could prove that subjective age is associated with body balance and muscle endurance. For instance, Li et al. [11], using a large sample of community-dwelling older people, found that adults with older subjective age than their chronological age were more likely to develop pre-frailty and frailty. It has been reported that body balance and muscle endurance, together with age and chronic diseases, are key risk factors for pre-frailty and frailty in the older population [29,30]. These studies indirectly confirm that an association exists between subjective age and body balance and muscle endurance.

Gait speed is a frequently used assessment tool and outcome measure in geriatric research and clinical settings. Gait speed has been considered as a predictor of functional decline and adverse health outcomes in older people [31,32]. The present study found that the felt younger participants had a faster walking speed compared to the felt older counterparts. Similarly, a large-sample study using linear regression analysis revealed that a younger subjective age than their chronological age was linked with faster gait speed in older persons [23]. Additionally, another previous study also documented that subjective age was connected to laboratory gait speed in older adults [33]. In light of these findings, it can be concluded that gait speed is also an indispensable physical fitness component that is closely related to subjective age in older people.

Cardiopulmonary endurance reflects the ability to perform an activity or exercise for long periods of time and generally refers to aerobic ability. Previous studies have documented that reduced cardiopulmonary endurance is related to an increased risk of developing functional decline and cognitive impairment in older people [34,35,36]. Functional decline and cognitive impairment have been reported to more or less be associated with subjective age [7,8,11]. This implies that there is a relationship between cardiopulmonary endurance and subjective age. The present study confirmed the above hypothesis. We used the 6 min walk test to evaluate cardiopulmonary endurance, finding that the 6 min walk distance was significantly associated with subjective age.

From a health perspective, much attention should be paid to older people who have an older subjective age than their chronological age due to a lower level of physical fitness. It is known that physical fitness is modifiable [37], and studies have shown that increased physical activity or exercise can enhance physical fitness [38,39,40,41,42]. Therefore, interventions to enhance physical fitness may be beneficial to improve the subjective age for older adults. Much attention should be paid to the promotion of physical fitness in improving the subjective age of older adults. Furthermore, longitudinal research is needed to determine the cause-and-effect relationship between physical fitness and subjective age in community-dwelling older adults.

This study has multiple limitations. First, the study’s findings were obtained based on community-dwelling older adults without cardiovascular diseases and musculoskeletal diseases. Thus, these findings may be inapplicable to other populations such as nursing home residents and unhealthy older people. Moreover, the study was a cross-sectional study, which does not allow us to reach any conclusions on the cause-and-effect association between physical fitness and subjective age. Third, subjective age was assessed by asking the participants a question in our study. This method has been challenged by several scholars, and they argued that the concept of subjective age cannot be assessed by a single question because of its multidimensionality [43,44,45]. Fourth, several confounding factors such as dietary nutrition, physical activity, and body composition were not considered in our study. These factors may have had an impact on the study results. Furthermore, although all the measurements of the participants were collected on the same day (9:00 a.m. to 5:00 p.m.), the time of day may influence the measurement results obtained by the participants. Finally, the detailed mechanisms of the association between physical fitness and subjective age were not clear. Future studies are needed to consider these limitations.

## 5. Conclusions

In summary, this study aimed to investigate the prospective relationship between subjective age and physical fitness and find out which aspects of the physical fitness components are closely related to subjective age, which augments our knowledge about physical fitness and subjective age. The findings suggest that subjective age is closely associated with physical fitness in community-dwelling older adults.

## Figures and Tables

**Table 1:** The basic characteristics of the participants.

	Older Men	Older Women	Overall
Numbers (n)	94	182	276
Chronological age (years)	71.21 ± 5.04	70.71 ± 4.82	70.88 ± 4.89
Subjective age (years)	69.09 ± 7.77	69.85 ± 7.11	69.59 ± 7.33
Age discrepancy (scores)	0.03 ± 0.06	0.01 ± 0.05	0.02 ± 0.05
Grip strength (kg)	37.20 ± 5.68	24.24 ± 4.34	28.66 ± 7.82
Balancing on one leg with eyes open (s)	43.09 ± 20.12	43.08 ± 22.47	43.08 ± 21.66
30 s chair stand test (repetitions/30 s)	20.91 ± 4.96	20.05 ± 5.44	20.35 ± 5.29
4 m habitual walk (s)	2.96 ± 0.46	3.05 ± 1.16	3.02 ± 0.98
6 min walk test (m)	605.50 ± 62.45	556.51 ± 84.65	573.19 ± 81.09

**Table 2:** The measured values of the physical fitness items across three different felt age groups in older men and women.

	① Felt Younger Group	② Felt the Same Group	③ Felt Older Group	*p* Values ① vs. ②	*p* Values ① vs. ③	*p* Values ② vs. ③
Older men						
Grip strength (kg)	39.98 ± 4.48	35.80 ± 5.61	33.83 ± 5.27	0.039	<0.001	0.746
Balancing on one leg with eyes open (s)	50.73 ± 17.50	37.82 ± 21.08	34.27 ± 19.53	0.125	<0.001	1.000
30 s chair stand test (repetitions/30 s)	23.92 ± 4.22	20.09 ± 4.32	17.06 ± 2.98	0.010	<0.001	0.071
4 m habitual walk (s)	2.80 ± 0.40	3.27 ± 0.44	3.08 ± 0.47	0.005	0.013	0.675
6 min walk test (m)	641.85 ± 37.53	598.27 ± 47.73	557.91 ± 61.95	0.028	<0.001	0.059
Older women						
Grip strength (kg)	26.79 ± 3.59	23.57 ± 3.89	22.42 ± 4.09	<0.001	<0.001	0.442
Balancing on one leg with eyes open (s)	56.37 ± 10.43	38.17 ± 24.65	34.14 ± 23.72	<0.001	<0.001	0.983
30 s chair stand test (repetitions/30 s)	23.79 ± 4.93	17.94 ± 4.51	17.85 ± 4.46	<0.001	<0.001	1.000
4 m habitual walk (s)	2.75 ± 0.41	3.12 ± 0.65	3.26 ± 1.61	0.374	0.020	1.000
6 min walk test (m)	613.77 ± 44.67	548.85 ± 97.92	512.35 ± 76.58	<0.001	<0.001	0.040

Note: Felt younger group includes participants with subjective age < chronological age; felt the same group includes participants with subjective age = chronological age; felt older group includes participants with subjective age > chronological age.

**Table 3:** The multiple linear regression analysis between the age discrepancy score and measured physical fitness items in older men and women.

	B	SE	Beta	t	*p* Values
Older men					
(Constant)	−0.257	0.045	-	−5.726	<0.001
Grip strength	0.003	0.001	0.247	3.974	<0.001
Balancing on one leg with eyes open	0.001	0.000	0.190	3.142	0.002
30-s chair stand test	0.005	0.001	0.387	5.666	<0.001
4-m habitual walk	−0.025	0.007	−0.202	−3.518	0.001
6-min walk test	0.001	0.000	0.268	4.044	<0.001
	R^2^ = 0.85, Adjusted R^2^ = 0.71, F = 46.35, *p* < 0.001				
Older women					
(Constant)	−0.206	0.023	-	-9.032	<0.001
Grip strength	0.003	0.001	0.295	5.270	<0.001
Balancing on one leg with eyes open	0.001	0.000	0.145	2.474	0.014
30-s chair stand test	0.003	0.001	0.356	6.053	<0.001
4-m habitual walk	−0.002	0.002	−0.050	−0.941	0.348
6-min walk test	0.001	0.000	0.211	3.376	0.001
	R^2^ = 0.54, Adjusted R^2^ = 0.53, F = 41.16, *p* < 0.001				

## Data Availability

The data presented in the study are available on request from the corresponding author.
